# PD108 Digital Transformation Of An Organization Supporting Informal Caregivers During Pandemic: Lessons Learned And Future Direction For A Sustainable Solution

**DOI:** 10.1017/S0266462324003507

**Published:** 2025-01-07

**Authors:** Amir J Khan, Ala Szczepura, Deidre Wild, Sara Nelson, Mark Collinson, Marc Greenwood, Sonja Woodhouse

## Abstract

**Introduction:**

The COVID-19 pandemic has catalyzed a move from face-to-face to online delivery of services by hospitals and primary care providers, but little is known about the impact of digital transformation in organizations supporting unpaid caregivers. The value of care provided by informal caregivers since the start of the COVID-19 pandemic has been estimated at EUR111 billion in England.

**Methods:**

The study assessed the impact of digital transformation in an English caregivers’ support organization covering a population of 0.98 million. A retrospective mixed method study was conducted of digital and non-digital support service utilization among caregivers in city and rural geographical areas from January 2019 to June 2021. Organizational performance and service quality indicators were compared for two financial years: 2019-2020 and 2020-2021. A survey of users was conducted to evaluate barriers to and facilitators of digital service uptake, computer proficiency among caregivers, and preferences for future digital service provision.

**Results:**

The number of caregivers registered with the organization rose by 36 percent to 20,237 in 2021. Monthly contacts rose by 225 percent to 6,500, with remote contacts rising from 65 to 85 percent. Observed behavior patterns differed between city and rural caregivers. Overall, one-to-one contacts increased by 89 percent and caregiver assessments by 21 percent, with no expansion in staffing. User-reported service quality improved in five out of eight indicators (p<0.05). The demographic characteristics of survey respondents (152 caregivers) were similar to all registered caregivers. The mean short form Computer Proficiency Questionnaire score of 25.61 indicated a relatively high computer proficiency. Qualitative analysis confirmed a preference for face-to-face and online options. The most highly rated online services were peer support groups and wellbeing assessment and support needs checks.

**Conclusions:**

Considering the economic importance of unpaid caregivers, more attention should be paid to the organizations supporting them and the potential for technology to enhance caregivers’ access to and benefit from such services. This initial assessment of digital transformation in one such organization demonstrates the potential for cost-effective service transition. Further research is required to inform sustainable future solutions.

